# Interactions of the Insulin-Like Growth Factor Axis and Vitamin D in Prostate Cancer Risk in the Prostate Cancer Prevention Trial

**DOI:** 10.3390/nu9040378

**Published:** 2017-04-12

**Authors:** Fayth L. Miles, Phyllis J. Goodman, Catherine Tangen, Kathleen C. Torkko, Jeannette M. Schenk, Xiaoling Song, Michael Pollak, Ian M. Thompson, Marian L. Neuhouser

**Affiliations:** 1 Department of Health Services, University of Washington, Seattle, WA 98105, USA; fmiles@fredhutch.org; 2 Cancer Prevention Program, Division of Public Health Sciences, Fred Hutchinson Cancer Research Center, Seattle, WA 98109, USA; pgoodman@fredhutch.org (P.J.G.); ctangen@fredhutch.org (C.T.); jschenk@fredhutch.org (J.M.S.); xsong2@scharp.org (X.S.); 3 Department of Pathology, University of Colorado, Anschutz Medical Campus, Aurora, CO 80045, USA; ktorkko@ucdenver.edu; 4 Departments of Medicine and Oncology, McGill University, Montreal, QC H3A 0G4, Canada; michael.pollak@mcgill.ca; 5 Cancer Therapy and Research Center, University of Texas Health Science Center, San Antonio, TX 78229, USA; ThompsonI@uthscsa.edu

**Keywords:** insulin-like growth factor, vitamin D, prostate cancer, odds ratio, nested case-control, logistic regression

## Abstract

Some, but not all, epidemiologic studies report an association between vitamin D and prostate cancer risk. The inconsistent findings might be explained in the context of modification by members of the insulin-like growth factor (IGF) axis. Data and specimens for this nested case-control study (*n* = 1695 cases and *n* = 1682 controls) are from the Prostate Cancer Prevention Trial (PCPT). Baseline serum samples were assayed for 25(OH)D, IGF-1, IGF-2, IGFBP-2, IGFBP-3, and the ratio of IGF1:BP3, along with insulin-related markers c-peptide and leptin. The presence of prostate cancer was assessed by prostate biopsy. Multivariate logistic regression was used to estimate odds ratios (OR) and 95% confidence intervals (CIs) for prostate cancer risk. There were no interactions between serum 25(OH)D and IGF analytes in relation to prostate cancer risk when PCPT treatment arms were combined. In the placebo arm, above median serum 25(OH)D levels were associated with increased risk of prostate cancer among men with higher IGF-2 (OR:1.33, 95% CI: 1.00–1.65), with a significant interaction between 25(OH)D and treatment arm (P_interaction_ = 0.04). Additionally, there was an interaction between treatment arm and serum IGFBP-3 (P_interaction_ = 0.03). Higher serum 25(OH)D may increase risk of prostate cancer in the presence of higher circulating IGF-2.

## 1. Introduction

Vitamin D (1,25(OH)_2_D) is a pleiotropic hormone affecting multiple biological processes including cellular differentiation, cell cycle control, gene transcription, and immunity, among others. The circulating metabolite 25-hydroxyvitamin D (25(OH)D) is hydroxylated to the active form, 1,25(OH)_2_D by cytochrome P-450-dependent enzymes. Previously, we reported an association of increased serum 25(OH)D with decreased risk of high-grade prostate cancer in the Prostate Cancer Prevention Trial (PCPT) [1]. 

The vitamin D receptor (VDR) is present in almost all tissues, and is expressed by prostate epithelial cells [2]. However, the role of vitamin D in prostate cancer etiology is unclear. Vitamin D has been shown to inhibit cell proliferation in animal models of prostate cancer as well as in culture [3,4]. Importantly, serum or plasma vitamin D has been shown to be inversely associated with prostate cancer risk in epidemiological studies [5,6], consistent with our findings [1]. However, other studies have shown positive [7,8] or null associations of vitamin D concentrations with prostate cancer risk [9,10,11,12,13,14].

The insulin-like growth factor (IGF) axis plays an important role in prostate cancer etiology and progression [15,16,17]. This axis is comprised of IGF-1, IGF-2, insulin, and their respective receptors, as well as binding proteins, which regulate many physiological processes. While IGF-1 and -2 are important for growth and development of normal and tumorigenic prostate epithelial cells, insulin-like growth factor binding proteins (IGFBPs) regulate the half-lives and bioavailability of IGFs for binding of their receptors [18,19]. Circulating IGF-1 has been associated with increased risk of advanced prostate cancer [20], and binding proteins may be associated with prostate cancer development or progression [21,22]. Previously, we examined levels of IGF axis analytes within the PCPT and reported an association of increased serum IGFBP-2 with prostate cancer risk in the placebo, but not the finasteride, arm of the study [23] .

Biological evidence suggests that the IGF axis may interact with vitamin D in carcinogenesis [24]. However, this interrelation is very complex and not well understood as both have a wide variety of effects that are cell- and context-specific. Vitamin D has been shown to increase serum levels of IGF-I and IGFBP-3. For example, serum 25(OH)D3 was positively correlated with serum IGF-1 (*r* = 0.33) in healthy adults, and oral supplementation of a single dose of 300,000 IU vitamin D increased levels of IGF-1 and IGFBP-3 in children with rickets [25,26]. On the other hand, IGF-1 increases the production of enzymes involved in vitamin D activation [27], and may stimulate synthesis of vitamin D [28,29]. Importantly, 25(OH)D and the IGF axis may cross talk during cancer-related signaling. For example, vitamin D was shown to antagonize the pro-tumorigenic effects of IGF-1 and stimulate overexpression of IGFBP-3, resulting in decreased proliferation of breast cancer cells [30,31]. Similarly, IGFBP-3 was found to mediate vitamin D-induced growth inhibition in LNCaP prostate cancer cells [32,33]. Most of these studies were conducted in cell cultures and in vitro systems; it is unknown if these same interactions occur in free living humans. It was for this reason that, using a nested case-control design in a substudy of the Prostate Cancer Prevention Trial (PCPT) designed to examine the ability of finasteride to prevent prostate cancer, we sought to investigate the interplay of the IGF axis and serum hydroxyvitamin D in prostate cancer risk. We examined both finasteride and placebo arms separately due to the previously-reported association of finasteride, an inhibitor of the conversion of testosterone, with reduced risk of low-grade prostate cancer. Our results indicate that the association of serum 25(OH)D with prostate cancer in the PCPT may be modified by both serum levels of IGF-2 and treatment arm.

## 2. Materials and Methods

### 2.1. Study Design and Population

Data for this nested case-control study are from the Prostate Cancer Prevention Trial (PCPT). 

The PCPT was a phase III randomized, placebo-controlled trial to test the effect of finasteride on primary prevention of prostate cancer. Details about the study design and primary trial results have been described elsewhere [34]. The study included 18,880 men over the age of 55 years enrolled in 221 clinical centers across the US with normal digital rectal exam (DRE) and prostate-specific antigen (PSA), and no history of prostate cancer or significant symptoms of benign prostate hyperplasia. Men underwent annual PSA and DRE screens; a biopsy was recommended in the event of an abnormal DRE or PSA above 4.0 ng/mL; all cancer-free subjects were recommended to undergo an end of study biopsy after seven years of on-study treatment. Examination of biopsies was performed by the pathologist at the local study site, as well as the central pathology laboratory. All participants signed written informed consent, and study procedures were approved by the Institutional Review Board at the Fred Hutchinson Cancer Research Center. Data on safety, adherence, and diagnosis of prostate cancer was reviewed every six months throughout the study by an independent data safety and monitoring committee. 

Cases (*n* = 1695) included men with biopsy-confirmed cancer of the prostate, identified during a for-cause biopsy after an abnormal DRE or elevated PSA or through an end-of-study biopsy, and for whom baseline serum samples were available. Prostate cancers of Gleason <7 were defined as low-grade, and those of Gleason ≥7 were defined as high grade. Controls (*n* = 1682) included men with no evidence of prostate cancer based on the end-of-study biopsy with available baseline serum samples, and all available non-White men. Cases and controls were frequency matched on age, race, family history of prostate cancer, and treatment arm. Men missing baseline serum 25(OH)D or IGF analytes were excluded from analyses. 

### 2.2. Data Collection

Non-fasting blood samples were collected at recruitment, approximately three months prior to randomization to the finasteride or placebo arm, and yearly thereafter. Samples were processed, aliquoted and stored at a central repository. Assays for 25(OH)D were performed by the Fred Hutchinson Cancer Research Center Public Health Sciences Biomarkers Laboratory as described previously [1]. Serum 25(OH)D was assayed with the LIAISON 25 OH Vitamin D TOTAL Assay (DiaSorin Inc.) following the manufacturer’s instructions. The lower limit of quantitation of this assay is 4 ng/mL. Assays for serum IGF-axis markers, IGF-1, IGF-2, IGFBP-2, and IGFBP-3, c-peptide, and leptin, were conducted at McGill University in the Jewish General Hospital (Montreal, Quebec, Canada). A standard ELISA was used to measure concentrations of IGF-1, IGF-2, IGFBP-2, IGFBP-3, as well as c-peptide and leptin in duplicate with inclusion of two sets of quality control samples, as described previously [23]. Laboratory personnel were blinded to the treatment arm and case status. 

Participants completed several standardized, self-administered questionnaires at baseline. Data were collected on demographic characteristics, first-degree relative family history of prostate cancer, medical history (including diabetes), and lifestyle habits including tobacco smoking, alcohol use, and usual physical activity [35]. Height and weight were measured at the baseline clinic visit. Body mass index (BMI) was calculated as weight (kg)/height (m^2^). Vitamin D and calcium intake along with alcohol intake were assessed one year after randomization using a food frequency questionnaire and structured dietary supplement use questionnaire [36]. 

### 2.3. Statistical Analysis

The overall goal of this analysis was to determine whether IGF axis analytes modified the association of serum 25(OH)D with prostate cancer risk, considering the finasteride and placebo arms combined, as well as separately. Concentrations of serum 25(OH)D were standardized for month of blood collection from participants using the residual method, whereby the residual from a model regressing month on 25(OH)D concentration was added to the population mean concentration (60.74 nmol/L). The ratio of IGF-1 to IGFBP-3 was obtained by dividing the concentration of serum IGF1 by IGFBP-3. 

All analyses were conducted using SAS (version 9.4, Cary, NC, USA). Unconditional logistic regression models were used to estimate multivariate adjusted odds ratios and 95% confidence intervals for associations of 25(OH)D with prostate cancer risk, after stratifying according to the above or below median concentrations of IGF-axis analytes. Models were adjusted for age, race (White vs. non-White), family history of prostate cancer, smoking (current, former, or non-smoker, and pack-years), and treatment arm (finasteride vs. placebo) where appropriate. Diabetes status, physical activity [37], education, and total dietary calcium (food + supplements) were also considered but found to be non-influential on the results and, thus, not included in the final models. Logistic regression was also used to estimate univariate odds ratios analyzing the association between serum 25(OH)D and IGF, with the latter as the outcome variable. All tests were two-sided and statistical significance was defined as *p* < 0.05. *p* values were not adjusted for multiple comparisons, given the limited number of analytes examined.

Finasteride and placebo arms were examined combined and separately. The association of serum 25(OH)D with prostate cancer according to IGF analyte-status was examined using the quartile distribution or median cut points for 25(OH)D, and median cut points for IGF axis analytes among controls. Cross-product interaction terms for serum 25(OH)D and treatment arm were included for analyses of modification by treatment arm on the association of 25(OH)D and prostate cancer risk, and a chi-square test was performed for heterogeneity. 

## 3. Results

Baseline characteristics of the nested case-control sample from the PCPT are shown in Table 1. No notable differences were observed between age, family history, and study arm due to the initial matching of these factors. Diabetes history was different between cases and controls (4.5% vs. 7.4%). Ethnicity differed between cases and controls due to the selection of the controls to include all available non-White men. A lower proportion of cases had high physical activity compared to controls (8.6% vs. 10.3%) and the average BMI was higher for cases than controls (27.4 vs. 26.6 kg/m^2^). A higher proportion of cases had college or post-graduate education (55.9% vs. 50.9%). However, none of these differences were statistically significant. 

Mean concentrations of serum 25(OH)D, IGF analytes, leptin, and c-peptide are shown in Table 2. There were no statistical differences between cases and controls, although the mean concentrations of all biomarkers, except leptin and the IGF1:IGFBP3 ratio, were slightly higher among cases.

To examine the association of serum 25(OH)D with IGF-analytes, we performed univariate analyses on these markers (Table 3). Serum 25(OH)D was positively associated with odds of above-median serum concentrations of IGF-1 (*p* for trend = 0.0004), and IGFBP-2 (*p* for trend = 0.0001). Serum 25(OH)D was inversely associated with c-peptide (*p* for trend = 0.0001) and leptin (*p* for trend = 0.0001) in a strong dose-response manner.

Next, we determined if IGF-axis analytes modify the association of 25(OH)D with overall prostate cancer risk, considering both study arms combined (Table 4). Overall, IGF axis analytes and related biomarkers did not alter the association of 25(OH)D with prostate cancer risk. The direction of the association between 25(OH)D and prostate cancer appeared to differ for strata of lower vs. higher IGF-2, but findings were not significant. There were no statistically significant interactions (data not shown). There were no associations with high or low grade prostate cancer after stratification according to IGF analytes. However, power to detect associations by grade was limited (Table S1, Supplementary).

We analyzed the association of serum concentrations of 25(OH)D with prostate cancer within strata of high and low serum concentrations of IGF analytes within the finasteride (Table S2, Supplementary) and placebo (Table S3, Supplementary) arms. No statistically significant associations were observed, although there was a general trend of an inverse association of 25(OH)D with prostate cancer risk in the finasteride arm but positive association in the placebo arm. 

Given the suggestion of differences in the association of 25(OH)D with prostate cancer by treatment arms among men with higher IGF analyte concentrations (Supplementary Tables S2 and S3), we tested whether treatment arm modified the association between 25(OH)D and prostate cancer among these men (Table 5). We found that among men with higher serum IGF-2 concentrations, the association of 25(OH)D with prostate cancer was modified by treatment arm (OR, finasteride: 0.84, 95% CI: 0.62–1.14; OR, placebo: 1.33, 95% CI: 1.00–1.65); *p* for interaction = 0.04). Additionally, the treatment arm modified the association of 25(OH)D with prostate cancer among individuals with higher serum IGFBP-3 (OR, finasteride: 0.83, 95% CI: 0.61–1.12; OR, placebo: 1.26, 95% CI: 0.98–1.63; *p* for interaction = 0.03). A marginal interaction was also observed for leptin, although associations were not statistically significant in either arm. (OR, finasteride: 0.76, 95% CI: 0.56–1.04; OR, placebo: 1.13, 95% CI: 0.87–1.47; *p* for interaction = 0.05). 

## 4. Discussion

The interaction of 25(OH)D with the IGF axis in the initiation or progression of prostate cancer has not been well studied. In the current study serum levels of these biomarkers were examined in prostate cancer cases and controls of the PCPT. Although there were no interactions detected between serum 25(OH)D and IGF analytes when considering combined treatment arms, 25(OH)D was associated with an increased risk of prostate cancer in the presence of higher serum IGF-2 (≥1707.59 ng/mL) in the placebo arm of the trial. Further evaluation revealed an interaction of the treatment arm with 25(OH)D among men with higher serum IGF-2, with a notable effect size.

Similar to IGF-1, IGF-2 is a potent mitogen and regulator of cell growth, differentiation, and metabolism, and is highly expressed during fetal development [38]. IGF-2 mRNA was increased in samples of patients with castrate resistant prostate cancer and promoted steroidogenesis resulting in activation of the androgen receptor in vitro [39]. We did not observe an increased risk of prostate cancer with higher serum IGF-2 in the PCPT when analyzed previously, in either finasteride or placebo treatment arms, although men in the highest quartile of serum IGFBP-2 had increased risk of total and low-grade prostate cancers [23].

Since there was a general trend towards a positive association of serum 25(OH)D with prostate cancer risk among men with higher levels of IGF analytes in the placebo arm, but a decreased risk among men in the finasteride arm, finasteride treatment and IGF concentration together may be important modifiers of the association of vitamin D with prostate cancer risk. The primary outcome of the finasteride intervention in the PCPT was a 25% reduction in prostate cancer compared to men randomized to placebo [34]. Our findings suggest a potential benefit of finasteride treatment that precludes an unfavorable interaction of IGF-2 and 25(OH)D. In the absence of finasteride treatment and under conditions of higher IGF-2, higher levels of serum 25(OH)D may increase prostate cancer risk. Our findings of modification of the association of serum 25(OH)D with cancer risk by IGF analytes may help to explain inconsistent findings of the association of serum 25(OH)D with prostate cancer. 

IGFBPs transport IGFs, increasing their stability and regulating their distribution, consequently limiting bioavailability [18]. IGFs, particularly IGF-1, are frequently sequestered by IGFBP-3 inhibiting mitotic effects [40], and may also be regulated by IGFBP-2 [19], in addition to other binding proteins. IGFBP-2 proteins may have IGF-independent roles as well, and may be associated with prostate cancer progression [22]. Previously, when we examined concentrations of IGF axis analytes in serum of subjects in relation to prostate cancer risk in the PCPT, IGFBP-2 was found associated with increased risk for prostate cancer in the placebo arm [23]. In the current study, IGFBP-2 did not modify the associations of serum 25(OH)D with prostate cancer in spite of the positive correlation between 25(OH)D and IGFBP-2 at baseline. However, the association with prostate cancer risk was stronger in the placebo arm than with finasteride, similar to our previous findings. 

As the majority of circulating IGF-1 is bound to IGFBP-3 [41], the IGF1:BP3 ratio may play a critical role in cancer progression. However, effects may differ depending on cancer type. Higher serum levels of IGF-1 and lower levels of IGFBP-3 have been associated with increased risk of aggressive prostate cancer [42] although higher levels of IGFBP-3 have been observed in prostate tumor cells compared to benign cells, and are associated with higher cancer recurrence [43]. In our previous analyses we did not find this ratio to be a predictor of prostate cancer risk among men in the PCPT [23]. However, in the current analysis there was an interaction of 25(OH)D and treatment arm among men with higher IGFBP-3, suggesting that 25(OH)D may increase the prostate cancer risk in men with higher levels of IGFBP-3 not receiving finasteride treatment. There were also modest associations of IGF1:BP3 and IGFBP-3 with serum 25(OH)D at baseline. It should be noted that a different binding protein, IGFBP-1, which is responsible for shuttling IGF across capillary membranes into the target tissue [44], might be more predictive of prostate cancer risk, though concentrations were not measured in this study. 

It was an unexpected finding that no interaction was observed between IGF-1 and serum 25(OH)D in this study, as we anticipated an association with increased risk of prostate cancer, given the role of IGF-1 in prostate carcinogenesis [45,46]. There is evidence that vitamin D may increase levels of IGF-1 in healthy individuals [26], and increased levels of IGF-1 in serum have been correlated with increased prostate cancer risk [20,47]. This is consistent with our findings of a strong positive correlation between 25(OH)D and IGF-1 serum concentrations.

We anticipated detection of an interaction between c-peptide, a biomarker for insulin, and 25(OH)D with prostate cancer, as we have found high levels of c-peptide to be associated with a near two-fold increased risk for high-grade prostate cancer [48]. Insulin may promote cell proliferation [49], exerting a pro-survival effect on cancer cells [50], and may increase inflammation and oxidative stress [51]. In addition, positive associations were observed between 25(OH)D and c-peptide at baseline. However, again, the limitations of sample size may have precluded detection of such an association. 

In addition to the IGF-axis, other interacting partners may influence the association of vitamin D with prostate cancer risk. Polymorphisms or miRNA regulation associated with the VDR could affect prostate cancer risk or PSA levels [52,53]. We did not find any significant associations of single nucleotide polymorphisms (SNPs) in the VDR with prostate cancer risk in the PCPT (manuscript in preparation). Furthermore, examination of the interaction of vitamin D-related SNPs with IGF-1 or -2 status (median-separated) in prostate cancer risk revealed no significant associations after correction for multiple comparisons (data was not shown).

Race is an independent predictor of vitamin D, and African American and Asian men in general have lower plasma 25(OH)D compared to White men, which might be associated with increased cancer risk [54,55,56]. In addition to modification by race, the association of 25(OH)D with prostate cancer was shown to be modified by the plant flavonoid quercetin, with increased dietary intake associated with increased risk for men with normal levels of 25(OH)D (>30 ng/mL) [56]. Therefore, it is not clear how micronutrients that potentially interact with the VDR might impact the association of serum 25(OH)D and prostate cancer in the context of increased serum IGF-axis proteins. 

A major strength of the PCPT was the end-of-study biopsy recommended to all undiagnosed men to either confirm a diagnosis of prostate cancer or its absence; other strengths of this study were annual prostate screenings of all subjects and a central pathology review for all cancer cases. Additional strengths of our study include the matching criteria which limited confounding, geographical diversity of the study population, and data collection on lifestyle factors including dietary and supplemented vitamin D and physical activity, and baseline collection of serum for biomarker analysis. Limitations of sample size may have precluded detection of stronger interactions between 25(OH)D, IGF axis analytes, treatment arm, and behavioral factors, and increased the possibility of spurious associations as a result of multiple comparisons. Additionally, we were unable to effectively analyze the relevance of the interaction of 25(OH)D in prostate cancer stage and grade due to the limited numbers of high-grade cancers. Moreover, concentrations of 25(OH)D and IGF analytes measured at baseline may not be indicative of long-term status as fluctuations might inevitably accompany lifestyle and other changes. The small number of African American men precluded further analysis by race and, thus, we could not perform sub-group analyses in African Americans. Lastly, there is a small possibility of sampling error associated with the end of study biopsy [57]. Although bias is minimized by the inclusion of only biopsy-confirmed cases and controls in this nested case-control study, there nonetheless can be residual bias if the decision for biopsy was associated with other factors.

IGF analytes may be regulated by various factors. Circulating IGF-1 may be increased by intake of animal protein, including meat, seafood, milk and diary, and some minerals, increased caloric intake, and insulin [58,59,60,61]. While not expected to have a large impact on results, the ability of diet to regulate IGF-axis proteins in serum is a noteworthy consideration in light of the use of non-fasting blood samples in the current study, which could contribute to residual confounding. 

Signaling by the IGF axis may be cell-specific and very complex as a result of cross-regulation and bioavailability. Physiological effects of any one analyte might only be interpreted in the context of serum levels and bioactivity of the other axis members. While we noted baseline associations of IGFs and binding proteins with serum 25(OH)D, the exact regulatory mechanism is unclear—whether 25(OH)D regulates IGFs or vice versa, and there could be additional players mediating the interaction. It is also possible that increased levels of IGFs are associated with tumor initiation, and serum 25(OH)D acts to promote the growth of an existing tumor or preneoplastic lesion. Further studies are warranted to determine the relationship between 25(OH) D and IGF-axis members.

Moreover, there may be very relevant thresholds to consider, dictating the biological consequences of 25(OH)D and IGF analytes, or their cross-regulatory ability. For example, in a study of the effects of the interaction of IGF-1 with 25(OH)D in metabolic syndrome, individuals with higher levels of both vitamin D and IGF-1 had lower prevalence of metabolic syndrome, but this benefit was only seen up to a 25(OH)D threshold of 75–85 nmol/L [62]. For this reason, we may have been unable to detect relevant interactions by considering only the median cut points. Furthermore, levels of IGF analytes and related biomarkers may be affected by genetic and lifestyle factors, which may modify the regulatory activity of these biomarkers. For example, increased vitamin D has been associated with a decline in IGF-1:BP3 ratio in obese individuals [63], suggesting that vitamin D promoted an imbalance in IGF-1:BP3 levels, which was affected by weight. In additional analyses, we determined if there was any modification of the 25(OH)D-IGF interaction by body mass index and smoking, but no statistical associations were detected. More research is necessary to ascertain the roles of these potential modifying factors in 25(OH)D-related prostate cancer risk. 

## 5. Conclusions

In conclusion, our findings indicate that increased serum 25(OH)D was associated with increased prostate cancer risk among men with higher circulating levels of IGF-2 in the placebo arm of the PCPT study. Although this was an exploratory analysis, the findings suggest that vitamin D may be associated with increased prostate cancer risk among men with higher IGF-2, and possibly other IGF analytes, in the absence of finasteride treatment. Our findings warrant further examination of the interaction of 25(OH)D, finasteride, and IGFs, and their respective binding proteins.

## Figures and Tables

**Table 1:** Baseline characteristics of cases and controls in the Prostate Cancer Prevention Trial.

	Cases	Controls
Number of participants	1695	1682
Arm, No. (%)		
Placebo	987 (58.2)	971 (57.7)
Finasteride	708 (41.8)	711 (42.3)
Age, mean, SD	63.7, 5.6	63.6, 5.6
Family history, No. (%)	366 (21.6)	362 (21.5)
History of diabetes No. (%)	77 (4.5)	125 (7.4)
Ethnicity, No. (%)		
White	1603 (94.6)	1436 (85.4)
Black	82 (4.8)	171 (10.2)
Other	10 (0.6)	75 (4.5)
Education, No. (%)		
High school	289 (17.1)	333 (19.8)
Some college	458 (27.0)	492 (29.25)
College/post-graduate	947 (55.9)	856 (50.9)
Alcohol intake, mean (SD) ^a^	9.8, 15.6	9.2, 13.8
Drinking status, No. (%)		
Nondrinker	381 (22.4)	392 (23.3)
>0–<30	1161 (68.5)	1142 (67.9)
≥30	153 (9.0)	148 (8.8)
Smoking, No. (%)		
Never	601 (35.5)	574 (34.13)
Current	115 (6.8)	124 (7.4)
Former	979 (57.8)	984 (58.5)
Pack-years, mean (SD)	14.2 (16.3)	15.2 (16.8)
BMI (Kg/m^2^), mean (SD)	27.4 (4.0)	26.6 (4.0)
Physical activity ^b^		
Light	989 (58.4)	990 (58.9)
Moderate	554 (32.7)	511 (30.4)
Active	145 (8.6)	173 (10.3)
Dietary Vitamin D, mean (SD) ^c^	62.0 (24.1)	59.4 (23.1)
Dietary Calcium, mean (SD) ^c^	1090 (596)	1049 (578)

^a^ Alcohol intake in grams per day; ^b^ Activity index calculated from frequency, duration, and pace; light = low activity index and intense activity <4/week; moderate = moderate or high activity index and intense activity ≤4 times/week; active = high activity index and intense activity ≥5 times /week; ^c^ Intake calculated as mg/day.

**Table 2:** Concentrations of serum biomarkers among cases and controls ^a^.

	Cases (*n* = 1695)	Controls (*n* = 1682)
Serum biomarker, mean (SD)		
25(OH)D (nmol/L)	62.1 (24.1)	59.4 (23.1)
IGF-1 (ng/mL)	211.3 (65.5)	210.2 (63.9)
IGF-2 (ng/mL)	1758.8 (434.3)	1733.0 (436.5)
IGFBP-2 (ng/mL)	557.4 (318.7)	511.1 (301.0)
IGFBP-3 (ng/mL)	4078.7 (969.2)	4035.5 (981.8)
IGF1:IGFBP3 (ng/mL)	0.05 (0.01)	0.05 (0.01)
C-peptide (ng/mL)	3.7 (2.3)	3.6 (2.3)
Leptin (ng/mL)	9.6 (7.2)	10.4 (8.0)

^a^ Serum samples collected at baseline.

**Table 3:** Univariate odds ratios depicting the association of serum 25(OH)D with IGF analytes ^a^.

IGF Axis Median Cutpoints (ng/mL)	25(OH)D nmol/L ^b^		N Upper Median/Lower ^c^	OR (95% CI)	*p* ^d^
**IGF-1 (204.78)**	Q1	42.92	311/420	1.0	
	Q2	42.93–56.40	425/424	1.35 (1.11–1.65)	
	Q3	56.41–72.37	491/419	1.58 (1.30–1.93)	
	Q4	>72.38	460/424	1.47 (1.20–1.79)	0.0004
**IGF-2 (1730.20)**	Q1	0–44.04	372/418	1.0	
	Q2	44.05–58.15	479/426	1.26 (1.04–1.53)	
	Q3	58.16–74.00	449/425	1.19 (0.98–1.44)	
	Q4	>74.01	386/418	1.04 (0.85–1.26)	0.92
**IGFBP-2 (461.90)**	Q1	0–41.30	240/422	1.0	
	Q2	41.31–54.03	340/423	1.41 (1.14–1.75)	
	Q3	54.04–67.63	403/418	1.70 (1.38 -2.09)	
	Q4	>67.64	704/423	2.93 (2.40–3.57)	0.0001
**IGFBP-3 (4033.35)**	Q1	0–43.79	360/421	1.0	
	Q2	43.80–57.52	449/419	1.25 (1.03–1.52)	
	Q3	57.53–72.87	441/424	1.22 (1.00–1.48)	
	Q4	>72.88	437/422	1.21 (1.0–1.47)	0.09
**IGF1:BP3 (0.05)**	Q1	0–44.04	369/421	1.0	
	Q2	44.05–57.02	400/420	1.09 (0.89–1.32)	
	Q3	57.03–72.37	457/423	1.23 (1.02– 1.49)	
	Q4	>72.38	461/422	1.25 (1.03–1.51)	0.01
**C-peptide (3.08)**	Q1	0–46.79	528/423	1.0	
	Q2	46.80–60.27	467/427	0.88 (0.73–1.05)	
	Q3	60.28–76.24	399/417	0.77 (0.64–0.93)	
	Q4	>76.25	293/419	0.56 (0.46–0.68)	0.0001
**Leptin (8.27)**	Q1	0–48.29	608/441	1.0	
	Q2	48.30–62.27	457/442	0.75 (0.63–0.90)	
	Q3	62.28–77.49	316/438	0.53 (0.44–0.64)	
	Q4	>77.50	229/442	0.36 (0.29–0.44)	0.0001

**^a^** Values for serum 25(OH)D and IGF-axis analytes measured at baseline; ^b^ Quartiles for serum 25(OH)D set using the distribution among men in the respective below median IGF category; ^c^ Number of men in the above median IGF category compared to those in the below median IGF category; ^d^ *p*-value for trend.

**Table 4:** Multivariate adjusted associations of serum 25(OH)D and overall prostate cancer risk according to serum levels of IGF axis analytes ^a,b^.

25(OH)D (nmol/L)		Cases/Controls	OR (95% CI)	25(OH)D nmol/L	Cases/Controls	OR (95% CI)
	**IGF-1 (<203.12)**			**IGF-1 (≥203.12)**		
Q1	(0–41.30)	161/210	1.0	(0–45.60)	182/208	1.0
Q2	(41.31–54.40)	200/209	1.06 (0.79–1.42)	(45.61–58.83)	231/209	1.15(0.87–1.52)
Q3	(54.41–69.88)	188/210	0.96 (0.71–1.28)	(58.84–73.06)	217/205	1.06 (0.80–1.52)
Q4	(≥ 69.89)	259/205	1.31 (0.98–1.74)	(≥73.07)	239/208	1.10 (0.83–1.46)
	**IGF-2 (<1720.22)**			**IGF-2 (≥1720.22)**		
Q1	(0–43.30)	189/210	1.0	(0–43.86)	163/208	1.0
Q2	(43.31–57.15)	189/210	0.83 (0.62–1.11)	(43.87–56.77)	229/208	1.28 (0.97–1.70)
Q3	(57.16–71.64)	188/206	0.81 (0.60–1.08)	(56.78–71.63)	234/209	1.27 (0.96–1.68)
Q4	(≥71.65)	243/207	1.02 (0.77–1.36)	(≥71.64)	241/206	1.26 (0.95–1.68)
	**IGFBP-2 (<443.40)**			**IGFBP-2 (≥443.40)**		
Q1	(0–39.43)	145/208	1.0	(0–47.91)	221/210	1.0
Q2	(39.44–52.41)	186/209	1.14 (0.84–1.53)	(47.92–61.14)	213/210	0.89 (0.68–1.75)
Q3	(52.42–66.88)	213/202	1.24 (0.92–1.67)	(61.15–76.24)	230/205	0.97 (0.74–1.27)
Q4	(≥66.89)	200/210	1.10 (0.82–1.49)	(≥76.25)	268/210	1.08 (0.83–1.41)
	**IGFBP-3 (<3999.39)**			**IGFBP-3 (≥ 3999.39)**		
Q1	(0–42.55)	173/210	1.0	(0–44.67)	182/210	1.0
Q2	(42.56–56.46)	194/210	0.96 (0.72–1.28)	(44.68–57.52)	220/206	1.10 (0.83–1.46)
Q3	(56.47–71.00)	194/207	0.93 (0.69–1.24)	(57.53–72.37)	230/206	1.12 (0.85–1.48)
Q4	(≥71.01)	237/207	1.10 (0.83–1.46)	(≥72.38)	246/208	1.14 (0.86–1.51)
	**IGF1:BP3 (<0.05)**			**IGF1:BP3 (≥0.05)**		
Q1	(0–42.67)	179/208	1.0	(0–44.92)	180/209	1.0
Q2	(42.68–55.28)	203/216	0.98 (0.81–1.20)	(44.93–58.27)	201/203	1.02 (0.83–1.24)
Q3	(55.29–70.81)	222/207	1.05 (0.86–1.28)	(58.28–72.62)	206/206	0.95 (0.78–1.16)
Q4	(≥70.8)	247/208	1.04 (0.85–1.26)	(≥72.63)	238/207	0.96 (0.79–1.17)
	**C-peptide (<3.01)**			**C-peptide (≥ 3.01)**		
Q1	(0–45.17)	151/213	1.0	(0–42.55)	202/211	1.0
Q2	(45.18–59.02)	206/203	0.82 (0.61–1.10)	(42.56–55.28)	220/211	1.05 (0.80–1.39)
Q3	(59.03–74.99)	218/208	0.81 (0.61–1.08)	(55.29–68.88)	193/206	1.21 (0.91–1.61)
Q4	(≥ 75.0)	217/210	0.86 (0.64–1.15)	(≥68.89)	269/202	0.87 (0.66–1.14)
	**Leptin (<8.57)**			**Leptin (≥8.57)**		
Q1	0–46.54	177/207	1.0	(0–40.42)	159/205	1.0
Q2	46.55–60.14	219/212	0.95 (0.72–1.26)	(40.43–53.72)	206/212	0.90 (0.67–1.20)
Q3	60.15–75.49	250/204	0.80 (0.61–1.06)	(53.73–67.32)	200/207	0.96 (0.71–1.28)
Q4	(≥ 75.50)	266/212	0.82 (0.62–1.08)	(≥67.33)	199/205	0.98 (0.73–1.31)

^a^ Models adjusted for age, race, BMI, treatment arm, and smoking; ^b^ Values for serum IGF-axis analytes measured in ng/mL and separated according to the median among controls.

**Table 5:** Odds ratios for the interaction of serum 25(OH)D with treatment arm in prostate cancer risk among individuals with elevated serum IGF axis analytes ^a,b^.

Finasteride			Placebo			
25(OH)D ^c^	cases/controls	OR (95% CI)	25(OH)D ^c^	cases/controls	OR (95% CI)	*p* ^d^
**IGF-1 (≥202.17)**			**IGF-1 (≥205.22)**			
<58.91	192/177	1.0	<58.78	217/238	1.0	
≥58.91	176/172	0.81 (0.60–1.10)	≥58.78	278/238	1.13 (0.87–1.47)	0.1
**IGF-2 (≥1722.33)**			**IGF-2 (≥1707.59)**			
<57.03	181/179	1.0	<57.16	226/245	1.0	
≥57.03	176/179	0.84 (0.62–1.14)	≥57.16	296/232	1.33 (1.00–1.65)	0.04
**IGFBP-2 (≥454.20)**			**IGFBP-2 (≥435.20)**			
<59.65	173/180	1.0	<61.53	255/245	1.0	
≥59.65	205/178	1.11 (0.83–1.49)	≥61.53	294/240	1.11 (0.87–1.43)	0.98
**IGFBP-3 (≥4001.50)**			**IGFBP-3 (≥3997.73)**			
<58.28	167/168	1.0	<57.16	209/232	1.0	
≥58.28	165/166	0.83 (0.61–1.12)	≥57.16	276/225	1.26 (0.98–1.63)	0.03
**IGF1:BP3 (≥0.05)**			**IGF1:BP-3 (≥0.05)**			
<58.59	176/176	1.0	<57.97	208/241	1.0	
≥58.59	179/172	0.92 (0.68–1.24)	≥57.97	268/239	1.15 (0.88–1.49)	0.27
**C-peptide (≥3.13)**			**C-peptide (≥2.92)**			
<54.54	171/175	1.0	<54.54	223/242	1.0	
≥54.54	185/172	0.93 (0.68–1.26)	≥54.54	296/236	1.25 (0.97–1.62)	0.13
**Leptin (≥8.58)**			**Leptin (≥8.60)**			
<54.16	169/175	1.0	<53.54	199/239	1.0	
≥54.16	150/174	0.76 (0.56–1.04)	≥53.54	243/236	1.13 (0.87–1.47)	0.05

^a^ Models adjusted for age, race, BMI, and smoking; ^b^ Serum IGF-2 and IGFBP-3 measured in ng/mL and separated by the median among controls; ^c^ Serum 25(OH)D measured in nmol/L and separated by the median among controls; ^d^ *p*-value for heterogeneity.

## Supplementary Materials

The following are available online at http://www.mdpi.com/2072-6643/9/4/378/s1, Table S1: Multivariate adjusted associations of serum 25(OH)D with risk of low or high grade prostate cancer according to serum levels of IGF axis analytes. Table S2: Multivariate adjusted associations of serum 25(OH)D with overall prostate cancer risk in the finasteride arm according to serum levels of IGF axis analytes. Table S3: Multivariate adjusted associations of serum 25(OH)D with overall prostate cancer risk in the placebo arm according to levels of IGF axis analytes.
