# Supplementary material for: Interactions of the Insulin-Like Growth Factor Axis and Vitamin D in Prostate Cancer Risk in the Prostate Cancer Prevention Trial
Source: Nutrients. 2017 Apr 12;9(4):378. doi: 10.3390/nu9040378 (PMC5409717; doi:10.3390/nu9040378)
Supplement: Supplementary file 1 [file nutrients-09-00378-s001.docx]

| **Supplementary Table S1.** Multivariate adjusted associations of serum 25(OH)D with risk of low or high grade prostate cancer according to serum levels of IGF axis analytes^1,2^. | | | | | | | | | | | | |
| --- | --- | --- | --- | --- | --- | --- | --- | --- | --- | --- | --- | --- |
|  |  |  | Low Grade | | | |  |  | High Grade | | | |
|  |  | **Lower Median (IGF axis)** | |  | **Upper Median (IGF axis)** | |  | **Lower Median (IGF axis)** | |  | **Upper Median (IGF axis)** | |
|  | **25(OH)D** | cases/ controls | OR (95% CI) |  | cases/ controls | OR (95% CI) |  | cases/ controls | OR (95% CI) |  | cases/ controls | OR (95% CI) |
|  |  |  |  |  |  |  |  |  |  |  |  |  |
| **IGF-1** | Lower | 227/419 | 1.0 |  | 279/417 | 1.0 |  | 116/419 | 1.0 |  | 122/417 | 1.0 |
|  | Upper | 300/415 | 1.14 (0.91–1.44) |  | 327/413 | 1.06 (0.85–1.31) |  | 127/415 | 1.02 (0.76–1.36) |  | 107/413 | 0.83 (0.62–1.12) |
|  |  |  |  |  |  |  |  |  |  |  |  |  |
| **IGF-2** | Lower | 238/420 | 1.0 |  | 266/416 | 1.0 |  | 121/420 | 1.0 |  | 113/416 | 1.0 |
|  | Upper | 295/413 | 1.07 (0.85–1.34) |  | 333/415 | 1.13 (0.91–1.40) |  | 119/413 | 0.92 (0.69–1.24) |  | 119/415 | 0.98 (0.73–1.32) |
|  |  |  |  |  |  |  |  |  |  |  |  |  |
| **IGFBP-2** | Lower | 214/417 | 1.0 |  | 291/420 | 1.0 |  | 104/417 | 1.0 |  | 125/420 | 1.0 |
|  | Upper | 280/412 | 1.13 (0.90–1.42) |  | 347/415 | 1.11 (0.90–1.37) |  | 116/412 | 1.03 (0.76–1.39) |  | 127/415 | 1.0 (0.75–1.32) |
|  |  |  |  |  |  |  |  |  |  |  |  |  |
| **IGFBP-3** | Lower | 231/420 | 1.0 |  | 272/416 | 1.0 |  | 119/420 | 1.0 |  | 117/416 | 1.0 |
|  | Upper | 290/414 | 0.99 (0.85–1.15) |  | 339/414 | 1.01 (0.87–1.18) |  | 120/414 | 0.96 (0.78–1.19) |  | 116/414 | 1.04 (0.84–1.29) |
|  |  |  |  |  |  |  |  |  |  |  |  |  |
| **IGF-1:BP-3** | Lower | 249/424 | 1.0 |  | 252/412 | 1.0 |  | 117/424 | 1.0 |  | 114/412 | 1.0 |
|  | Upper | 321/415 | 1.18 (0.94–1.46) |  | 310/413 | 1.06 (0.85–1.32) |  | 128/415 | 1.06 (0.79–1.42) |  | 113/413 | 0.90 (0.67–1.22) |
|  |  |  |  |  |  |  |  |  |  |  |  |  |
| **C-peptide** | Lower | 244/416 | 1.0 |  | 265/422 | 1.0 |  | 96/416 | 1.0 |  | 141/422 | 1.0 |
|  | Upper | 311/418 | 1.11 (0.89–1.38) |  | 312/408 | 1.06 (0.85–1.32) |  | 103/418 | 0.98 (0.72–1.35) |  | 132/408 | 0.89 (0.67–1.18) |
|  |  |  |  |  |  |  |  |  |  |  |  |  |
| **Leptin** | Lower | 267/419 | 1.0 |  | 226/417 | 1.0 |  | 109/419 | 1.0 |  | 128/417 | 1.0 |
|  | Upper | 364/416 | 1.24 (1.00–1.53) |  | 275/412 | 1.07 (0.85–1.34) |  | 126/416 | 1.07 (0.80–1.44) |  | 106/412 | 0.79 (0.59–1.06) |
| ^1^ Models adjusted for age, race, BMI, treatment arm, and smoking | | | | | | | | | | | | |
| ^2^ Concentrations of IGF axis analytes and 25(OH)D were separated according to the median among controls | | | | | | | | | | | | |

| **Supplementary Table S2.** Multivariate adjusted associations of serum 25(OH)D with overall prostate cancer risk in the finasteride arm according to serum levels of IGF axis analytes^a,b,c^. | | | | | | | |
| --- | --- | --- | --- | --- | --- | --- | --- |
|  |  | **Lower Median** | |  |  | **Upper Median** | |
| IGF axis median cutpoints (ng/mL) | 25(OH)D (nmol/L) | Cases/ Controls | OR (95% CI) |  | 25(OH)D (nmol/L) | Cases/ Controls | OR (95% CI) |
|  |  |  |  |  |  |  |  |
| **IGF-1** (202.17) | <53.54 | 146/179 | 1.0 |  | <58.91 | 192/177 | 1.0 |
|  | >53.54 | 186/176 | 1.04 (0.76–1.43) |  | >58.91 | 176/172 | 0.81 (0.60–1.09) |
|  |  |  |  |  |  |  |  |
| **IGF-2** (1722.33) | <56.28 | 161/173 | 1.0 |  | <57.03 | 181/179 | 1.0 |
|  | >56.28 | 182/173 | 0.90 (0.66–1.23) |  | >57.03 | 176/179 | 0.82 (0.61–1.11) |
|  |  |  |  |  |  |  |  |
| **IGFBP-2** (454.20) | <53.16 | 151/174 | 1.0 |  | <59.65 | 173/180 | 1.0 |
|  | >53.16 | 171/172 | 0.89 (0.64–1.23) |  | >59.65 | 205/178 | 1.09 (0.81–1.47) |
|  |  |  |  |  |  |  |  |
| **IGFBP-3** (4001.50) | <55.04 | 153/176 | 1.0 |  | <58.28 | 167/168 | 1.0 |
|  | >55.04 | 182/172 | 1.00 (0.73–1.39) |  | >59.65 | 165/166 | 0.80 (0.59–1.08) |
|  |  |  |  |  |  |  |  |
| **IGF-1:BP-3** (0.05) | <54.23 | 157/179 | 1.0 |  | <58.59 | 176/176 | 1.0 |
|  | >54.23 | 188/177 | 0.98 (0.72–1.34) |  | >58.59 | 179/172 | 0.88 (0.64–1.19) |
|  |  |  |  |  |  |  |  |
| **C-peptide** (3.13) | <59.03 | 168/179 | 1.0 |  | <54.54 | 171/175 | 1.0 |
|  | >59.03 | 176/178 | 0.90 (0.66–1.22) |  | >54.54 | 185/172 | 0.88 (0.65–1.20) |
|  |  |  |  |  |  |  |  |
| **Leptin**  (8.58) | <59.22 | 164/179 | 1.0 |  | <54.16 | 169/175 | 1.0 |
|  | >59.22 | 217/176 | 1.17 (0.87–1.58) |  | >54.16 | 150/174 | 0.71 (0.51–0.97) |
| ^a^ Models adjusted for age, race, BMI, and smoking. | | | | | | | |
| ^b^ Low and high values for serum IGF analytes and 25(OH)D separated by the median among controls. | | | | | | | |
| ^c^ Serum 25(OH)D measured in nmol/L; IGF axis analytes, c-peptide, and leptin measured in ng/mL. | | | | | | | |

| **Supplementary Table S3.** Multivariate adjusted associations of serum 25(OH)D with overall prostate cancer risk in the placebo arm according to serum levels of IGF axis analytes^a,b,c^. | | | | | | | |
| --- | --- | --- | --- | --- | --- | --- | --- |
|  |  |  |  |  |  |  |  |
|  |  | **Lower Median** | |  |  | **Upper Median** | |
| IGF axis median cutpoints (ng/mL) | 25(OH)D (nmol/L) | Cases/ Controls | OR (95% CI) |  | 25(OH)D (nmol/L) | Cases/ Controls | OR (95% CI) |
|  |  |  |  |  |  |  |  |
| **IGF-1** (205.22) | <55.54 | 217/245 | 1.0 |  | <58.78 | 217/238 | 1.0 |
|  | >55.54 | 265/239 | 1.16 (0.90–1.51) |  | >58.78 | 278/238 | 1.19 (0.92–1.53) |
|  |  |  |  |  |  |  |  |
| **IGF-2** (1707.59) | <61.15 | 245/269 | 1.0 |  | <57.16 | 226/245 | 1.0 |
|  | >61.15 | 207/213 | 0.99 (0.76–1.28) |  | >57.16 | 296/232 | 1.31 (1.02–1.68) |
|  |  |  |  |  |  |  |  |
| **IGFBP-2** (435.20) | <52.29 | 182/237 | 1.0 |  | <61.53 | 255/245 | 1.0 |
|  | >52.29 | 245/238 | 1.24 (0.95–1.62) |  | >61.53 | 294/240 | 1.14 (0.89–1.45) |
|  |  |  |  |  |  |  |  |
| **IGFBP-3** (3997.73) | <57.22 | 169/200 | 1.0 |  | <57.16 | 209/232 | 1.0 |
|  | >57.22 | 291/283 | 1.09 (0.83–1.43) |  | >57.16 | 276/225 | 1.29 (1.0–1.66) |
|  |  |  |  |  |  |  |  |
| **IGF-1:BP-3** (0.05) | <56.41 | 218/241 | 1.0 |  | <57.97 | 208/241 | 1.0 |
|  | >56.41 | 282/239 | 1.24 (0.96–1.60) |  | >57.97 | 268/239 | 1.19 (0.91–1.54) |
|  |  |  |  |  |  |  |  |
| **C-peptide** (2.92) | <59.53 | 201/241 | 1.0 |  | <54.54 | 223/242 | 1.0 |
|  | >59.53 | 256/241 | 1.18 (0.91- 1.53) |  | >54.54 | 296/236 | 1.28 (0.99–1.65) |
|  |  |  |  |  |  |  |  |
| **Leptin** (8.60) | <60.96 | 238/243 | 1.0 |  | <53.54 | 199/239 | 1.0 |
|  | >60.96 | 296/242 | 1.19 (0.93–1.51) |  | >53.54 | 243/236 | 1.16 (0.89–1.51) |
| ^a^ Models adjusted for age, race, BMI, and smoking. | | | | | | | |
| ^b^ Low and high values for serum IGF axis markers and 25(OH)D separated by the median among controls. | | | | | | | |
| ^c^ Serum 25(OH)D measured in nmol/L; IGF axis analytes, c-peptide, and leptin measured in ng/mL. | | | | | | | |
